# Fatigue database of additively manufactured alloys

**DOI:** 10.1038/s41597-023-02150-x

**Published:** 2023-05-02

**Authors:** Zian Zhang, Zhiping Xu

**Affiliations:** Tsinghua University, Applied Mechanics Laboratory and Department of Engineering Mechanics, Beijing, 100084 China

**Keywords:** Metals and alloys, Mechanical engineering, Mechanical properties, Databases, Databases

## Abstract

Fatigue is a process of mechanical degradation that is usually assessed based on empirical rules and experimental data obtained from standardized tests. Fatigue data of engineering materials are commonly reported in *S*-*N* (the stress-life relation), *ε*-*N* (the strain-life relation), and d*a*/d*N*-Δ*K* (the relation between the fatigue crack growth rate and the stress intensity factor range) data. Fatigue and static mechanical properties of additively manufactured (AM) alloys, as well as the types of materials, parameters of AM, processing, and testing are collected from thousands of scientific articles till the end of 2022 using natural language processing, machine learning, and computer vision techniques. The results show that the performance of AM alloys could reach that of conventional alloys although data dispersion and system deviation are present. The database (FatigueData-AM2022) is formatted in compact structures, hosted in an open repository, and analyzed to show their patterns and statistics. The quality of data collected from the literature is measured by defining rating scores for datasets reported in individual studies and through the fill rates of data entries across all the datasets. The database also serves as a high-quality training set for data processing using machine learning models. The procedures of data extraction and analysis are outlined and the tools are publicly released. A unified language of fatigue data is suggested to regulate data reporting for the fatigue performance of materials to facilitate data sharing and the development of open science.

## Background & Summary

**Fig. 1:**
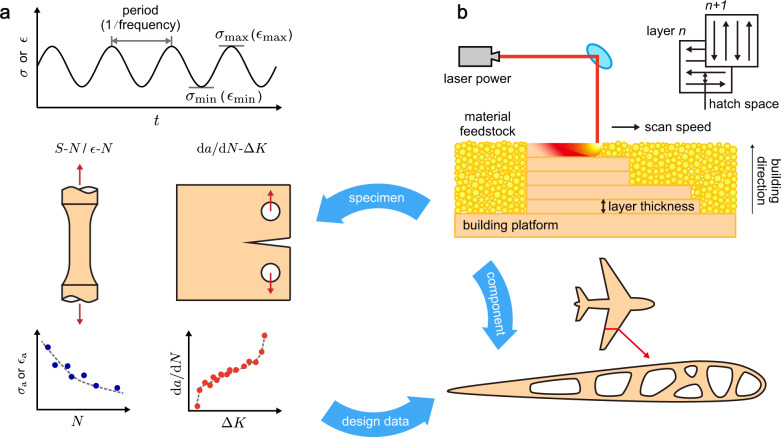
Structure integrity design of additively manufacturing (AM) structural components using fatigue data from standardized tests. (**a**) Representative loading conditions, types of specimens, and data obtained from fatigue tests. (**b**) Procedures and parameters of AM illustrated through the laser powder bed fusion (L-PBF) technique.

Fatigue is a detrimental process of mechanical degradation experienced by structural materials and components under long-term service in, for example, the aerospace, nuclear power, oil, and gas industry^1^. The design of structural integrity with the fatigue damage taken into account can be carried out in principles of safe life or damage tolerance. In safe-life design, flaws are not explicitly considered and products are intended to be removed from service after the design life. The philosophy of design relies on experimental data from standard specimens tested under specific loading conditions, which can be extended to structural components. In practice, arbitrary loading spectra are handled by considering cumulative damage, for example, by using the linear Miner’s rule^2^. The effects of the size of specimens, mean stress, multiaxiality, and environment can also be included. The stress-life (*S*-*N*) data produced by stress-controlled (force-controlled) tests and strain-life (*ε*-*N*) data by strain-controlled tests are the two fundamental sets of experimental data for safe-life design, which describe the relationship between the maximum (*σ*_max_, *ε*_max_) or amplitude (*σ*_a_, *ε*_a_) of stress/strain and the number of loading cycles (*N*) and are commonly used for high-cycle fatigue (HCF)/low-cycle fatigue (LCF) design, respectively (Fig. 1a). In damage-tolerance design, a structural component is considered to be able to sustain flaws (e.g. cracks) safely before the next inspection point, and the component is then repaired or replaced^2^. Fatigue crack growth (FCG) can be rationalized in the theory of fracture mechanics and experimentally assessed using compact-tension (CT) specimens. The dependence of the FCG rate (d*a*/d*N*) on the stress intensity factor (SIF) range (Δ*K*) is thus referred to in structural health monitoring and maintenance (Fig. 1a). The *S*-*N*, *ε*-*N* and d*a*/d*N*-Δ*K* data offer standard measures for the degradation of mechanical resistance under cyclic loads, which is a unique feature that can be exploited in data-centric research.

Compared to Young’s modulus and tensile strength, the fatigue performance of materials is susceptible to their microstructures, surface conditions as well as the loading and environmental conditions^2,3^. The fatigue process involves microstructural evolution from nano-, micro- to structural scales, and theoretical prediction of the performance remains challenging^4^. Fatigue databases thus become of crucial importance for structural design. The initiation of the Aircraft Structural Integrity Program (ASIP) in the 1950s led to great success in preventing catastrophic failures and prolonging the life of structural components. However, only a few databases are publicly released, usually by authoritative research institutions for conventional alloys, and are limited in types of materials and the number of data records. For example, the Metallic Materials Properties Development and Standardization (MMPDS) handbook includes 213 *S*-*N*, 15 *ε*-*N*, and 39 d*a*/d*N*-Δ*K* figures for 62 types of metallic materials, which are accepted for use in the Federal Aviation Administration (FAA), Department of Defense (DoD), and National Aeronautics and Space Administration (NASA)^5^. The National Institute for Materials Science (NIMS) Fatigue Data Sheet beginning in 1978 in Japan hosts 126 sheets of fatigue properties for 59 types of metallic materials^6^.

Standardized specimen preparation and testing conditions suppress most of the external sources of uncertainties in fatigue data and retain much of the correlation between the material performance and the material types as well as loading and environmental conditions. The reported fatigue data, however, still show a highly scattered nature for the variations in the microstructures of materials. From a complementary perspective, statistical analysis of this scattered nature based on a large volume of data may offer key insights into the material performance that cannot be reached by other means^7^.

Additive manufacturing (AM) is a facile technique to fabricate structural components with flexibility in structural design and benefits in the cost and lead time^8^ (Fig. 1b). Microstructural control offers an excellent route to explore the processing-microstructures-performance (PMP) relationship^9,10^. In the past few decades, significant efforts have been made to explore the performance limits of AM alloys, especially on their fatigue behaviors^11^. It is well-known that the surface conditions, internal defects, and other microstructural features strongly affect the fatigue performance of AM alloys, but the understanding of the PMP relationship remains largely qualitative^12,13^. Both physics-^14,15^ and machine learning (ML)-based approaches^16,17^ were developed to resolve this issue, which demands reliable fatigue data for model verification and validation (V&V). Although the volume of data is much smaller than that reported for alloys produced by conventional techniques such as casting and forging, thousands of papers have been published on the fatigue performance of AM alloys, which provide a complete subset of data for analysis. Recent studies collected and analyzed AM fatigue data of selected AM alloys (e.g. Ti-6Al-4V, AlSi10Mg/AlSi7Mg, 316 L) from the literature^18–21^. However, no datasets were released for follow-up data processing and analysis. Moreover, the quality of the summarized results is limited by the specific scope of the studies, and there is a need for standards or norms to report the fatigue performance of materials.

Open science, including open publication, data, and related resources, has recently become a global consensus to accelerate scientific research, promoting collaboration and benefiting the community^22,23^. Digitization and open-access development offer entirely new opportunities for data-centric studies based on literature data, which can be compiled into structured databases and used in, for example, material screening and engineering design. Compared to the data released by authoritative institutions, open data has its richness in the material microstructures and the conditions of testing, which may be helpful for gaining more insights into the PMP correlation. However, data heterogeneity is expected at least in the quality of test specimens and the design of fatigue tests, which should be assessed to produce reliable records. Journal articles, conference proceedings, and technical reports form a vast and continually growing corpus of unstructured information, which can be processed by state-of-the-art natural language processing (NLP), ML, and computer vision (CV) techniques. Progress has been witnessed in this direction, where databases for material synthesis recipes^24^ and properties^25–27^ were released.

In this work, we collect fatigue data and related data reported for AM alloys including titanium, nickel, aluminum, and steel from 3,415 scientific articles (up to the end of 2022). Open-source and in-house codes are used for data extraction from figures, tables, and text. The description of research and reported *S*-*N*, *ε*-*N* and d*a*/d*N*-Δ*K* data are outlined. To illustrate the usage of data, the fatigue performance of AM alloys is analyzed, offering suggestions for future research and more effective data publications.

## Methods

**Fig. 2:**
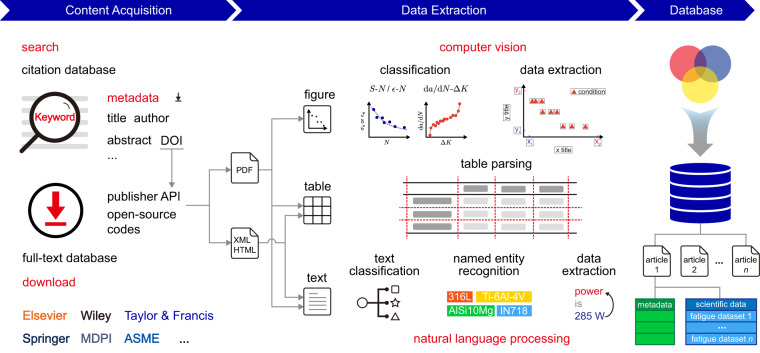
Workflow to construct the fatigue database of AM alloys. AM articles are searched on the Web of Science (WoS) and accessed via their digital object identifiers (DOIs). Types of materials, parameters of AM, processing, testing, as well as static mechanical and fatigue properties are extracted from figures, tables, and text, and structured into a hierarchical database.

Our workflow includes content acquisition (search and download), data extraction (from figures, tables, and text), and database construction (Fig. 2). The database contains metadata of articles and scientific data. Metadata includes information such as authors, funding agencies, and the year of publication, which outline the history of development, the state of the art, and the science of science (SciSci)^28^. Scientific data describes the contents of research such as the types of materials, parameters of AM, processing and testing, fatigue and static mechanical properties, and their relationship. The scientific data in each article are organized into separated fatigue datasets for the *S*-*N*, *ε*-*N* or d*a*/d*N*-Δ*K* data.

### Content acquisition

Articles focusing on AM fatigue are identified in the citation databases and the full text are downloaded from the publishers. Keywords for AM fatigue are summarized and compiled into search formulas (Table 1). In materials science or mechanics of materials, ‘fatigue’ generally covers studies on the behaviors under cyclic loads and is used as the search keyword. For AM, a series of synonyms, branches, and their abbreviations are used, according to the terminology found in the AM standards^29,30^ and review articles^31–35^. The search is conducted in the authoritative citation database, Web of Science Core Collection (WoS), through the fields of ‘title’, ‘abstract’, and ‘author keywords’. WoS returns 3,415 records of articles and their metadata are obtained through the ‘export’ function. An NLP model is applied for the classification of articles according to their abstracts^36^. Articles such as those on physiological ‘fatigue’ or research topics in irrelevant fields are discarded. Following NLP classification and manual examination, 2,001 candidate articles are identified.

**Table 1:** Keywords used for article search in the citation databases.

Category	Keyword
Fatigue	fatigue
Additive manufacturing	additive manufacturing/3D printing/selective laser melting/SLM/selective laser sintering/SLS/direct metal laser sintering/DMLS/electron beam melting/EBM/direct metal deposition/DMD/powder bed fusion/PBF/laser engineered net shaping/LENS/rapid prototyping/wire-arc additive manufacturing/WAAM/directed energy deposition/DED/laser metal deposition/LMD/laser solid forming/LSF/free-form fabricating/binder jetting/metal extrusion

The digital object identifiers (DOIs) in the metadata provide links to the full text. 104 of the 2,001 AM fatigue articles do not have DOIs in WoS records. In addition, 22 articles are not written in English, and 27 articles are from publishers with less than 10 publications. These records are discarded. 1,848 articles are downloaded for analysis and used to construct the database. Studies on the fatigue performance of AM alloys started after the year 2000, and most of the articles are published in both the portable document format (PDF) and extensible markup language (XML)/hypertext markup language (HTML) formats. PDF and XML/HTML files are more friendly to manual examination and automated code parsing, respectively. For Elsevier, 1,122 PDFs of articles are retrieved through the Application Programming Interface (API), accounting for 60% of the downloaded AM fatigue articles. PDFs from other sources are retrieved through the code article-downloader^37^ (24%), Scopus Document Download Manager (12%) or manually from the publishers’ sites (4%). Elsevier API provides access to XML files (60% articles). HTML files, if available, are retrieved from other publishers by using the code article-downloader (37% articles).

### Figure processing

The fatigue data (*S*-*N*, *ε*-*N*, and d*a*/d*N*-Δ*K*) presented as scatter plots in figures or entries in tables are extracted and stored as data pairs. Scatter plots are more readable and concise than tables and are widely adopted in the literature, although the latter presentation provides direct numerical values. Figures are extracted from the PDF documents using PyMuPDF. Figures containing fatigue data are screened and those with multiple plots are manually segmented into single plots. Scattered data points are extracted by an in-house MATLAB code IMageEXtractor (IMEX). The code enables automatic and manual data extraction and allows subsequent manual correction. The automatic extraction function includes axis calibration, legend parsing, and data recognition by employing CV techniques.

**Fig. 3:**
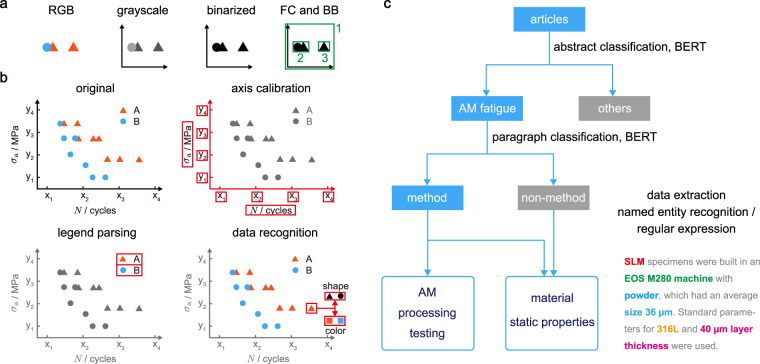
Figure and text data extraction. (**a**) RGB color figures are converted to grayscale and then binarized figures, where clusters of connected black pixels are detected as figure components (FCs). Their bounding boxes (BBs) are shown by green boxes. (**b**) The axes are detected in the figures and the legends are parsed to obtain the data symbols and labels. The symbols from the legends are used as templates for data recognition. Red boxes in each panel indicate the objects to be recognized in the steps of data processing. (**c**) Flowchart of text data classification and extraction.

The figures (98% published in color) are pre-processed into grayscale images and binarized by using a grayscale threshold of 80% to improve the efficiency of image processing in automatic extraction (Fig. 3a). The color, grayscale, and binarized versions of the figures are stored and selected for use in specific conditions. Clusters of connected black pixels in the binarized images are found and stored as figure components (FCs). The bounding box (BB) of an FC is defined as a rectangular region defined by its leftmost, rightmost, topmost, and bottommost pixels (Fig. 3a).

Axis calibration outputs the axis positions, axis labels, axis scales, ticks, and tick labels. The *x*-*y* coordinate system (CS) constitutes the largest FC, measured by the area under its BB. The *x*- and *y*-axis are identified as lines longer than 70% of the figure by scanning the largest FC in the vertical and horizontal directions. Lines perpendicular to axes are recognized as ticks. The labels are extracted by optical character recognition (OCR)^38^ and assigned to the axes and ticks according to their positions. The scales of axes (linear/log) are determined according to the position and label of ticks.

The legend regions are selected manually in the current study since the positions and layouts of legends vary from figure to figure. In the selected region, symbols of data points are recognized and stored as templates, and the legend labels are marked down. Pixels containing data points in the CS are recognized according to the color codes of templates. Data reported in the binarized representation are recognized using the shapes. In 55% of the d*a*/d*N*-Δ*K* data, the symbols are densely arranged and their shapes cannot be distinguished. Consequently, only pixels extracted using the color codes are stored. All *S*-*N*, *ε*-*N* and the rest 45% of d*a*/d*N*-Δ*K* data are extracted according to both color and shape that are consistent. The extracted pixels are matched to the shapes of templates to detect the types of symbols. The centroids of these symbols are then extracted as data points. The method of data extraction (‘color and shape’, ‘color’, or ‘shape’) is recorded in the database. The extracted axes, legends, and data are visualized and manually corrected in IMEX. Data extracted from figures are converted from pixel units to physical units according to the position and scale of ticks. Ticks at two ends of the axes are chosen as references to minimize the error in determining the locations.

The performance of figure data extraction can be assessed by the metrics

1 $${\rm{precision}}=\frac{{\rm{TP}}}{{\rm{TP}}+{\rm{FP}}},$$

2 $${\rm{recall}}=\frac{{\rm{TP}}}{{\rm{TP}}+{\rm{FN}}},$$

3 $${\rm{F}}1=2{\rm{}}\frac{{\rm{precision}}\times {\rm{recall}}}{{\rm{precision}}+{\rm{recall}}},$$

where TP denotes the true positive or the number of correctly-extracted data, FP is the false positive or the number of incorrectly-extracted data, and FN is the false negative or the number of data that are not extracted. The F1 score is the harmonic mean of precision and recall. The metrics of axis calibration, legend parsing, and data recognition are summarized in Table 2. We find that data recognition underperforms axis calibration and legend parsing due to the technical difficulties in analyzing overlapped data points.

**Table 2:** Evaluation metrics of automated data processing.

Source	Function	Precision	Recall	F1
figure	axis calibration	98%	96%	97%
	legend parsing	85%	97%	91%
	data recognition	82%	51%	63%
table	data extraction	52%	73%	60%
text	abstract classification	87%	93%	90%
	paragraph classification	87%	78%	82%
	data extraction	58%	68%	63%

### Table processing

Fatigue data in fewer than 5% articles are reported in tables. Tables are thus used in this work only to verify the data extracted from figures. Tables containing parameters of AM, processing, testing as well as static mechanical and fatigue properties are of interest, which can be identified from the table captions. Tables in XML/HTML files are parsed by table extractor^39^ whereas those embedded in the PDFs are processed manually. The evaluation metrics of table data extraction are summarized in Table 2. The F1 score is 60%, which is not high since the data of non-AM alloys or data from external references are included. Combining text information in processing data in the tables could improve performance.

### Text processing

Text processing includes text classification and data extraction (Fig. 3b). Structured text files in the XML/HTML format are processed using our in-house parsing codes TEXTract (adapted to the standard styles provided by the publishers) and in combination with the Python packages xml.dom.minidom for XML and BeautifulSoup for HTML. Text is extracted from PDFs by PDFDataExtractor^40^ if the XML/HTML files are not available.

Text classification is conducted for abstracts and paragraphs using the NLP library Simple Transformer. The Robustly Optimized BERT Pretraining Approach (RoBERTa)^36^, an improved model of the pre-trained Bidirectional Encoder Representation from Transformers (BERT)^41^, is used to transform text sequences into embedding vectors of abstract or paragraphs. The embedding vectors are passed to a fully connected neural network with one linear layer and output neurons corresponding to class labels. The RoBERTa and classification models are integrated into a classification module in Simple Transformer. The model is trained on AM fatigue articles with the AdamW^42^ optimizer using a cross-entropy loss function and a learning rate of 4 × 10^−5^. Abstract classification identifies AM fatigue articles from the search outputs of WoS based on a manually-labeled dataset of 500 abstracts, with class labels of ‘AM fatigue’ and ‘Non-AM fatigue’. Paragraphs are classified into ‘Method’ and ‘Non-method’ classes and passed to data extraction. ‘Method’ paragraphs include information of materials, parameters of AM, processing, and testing. The training set consisting of 3,350 paragraphs from 82 articles is constructed from sections with keywords of ‘method’, ‘fabrication’, ‘process’, ‘test’, and ‘experiment’ in their headings. Both abstract and paragraph datasets are split into training/testing/validation sets with a ratio of 0.8:0.1:0.1.

Data including the types of materials, parameters of AM, processing, testing, and static mechanical properties are extracted from text. To identify the types of materials, the chemical named entity recognition (NER) of ChemDataExtractor 2.0^43^ is applied together with a dictionary of the trade name of alloys, prepared according to MMPDS-17^5^ and the domain knowledge. The scope of AM materials recognition contains title, abstract, and method paragraphs. For data entries of AM, processing, and testing, keywords are summarized and organized into regular expressions (REs) to extract data from the ‘Method’ paragraphs. In a specific domain such as AM fatigue, where the variants of keywords and sentence patterns for target data are limited, it is relatively easy to construct the REs. In practice, one physical quantity may be associated with several data entries. For example, ‘temperatures’ are relevant for specifications of AM procedures, heat treatment, and fatigue testing. Therefore, the extracted data are assigned to entries according to manually defined keywords in the current and previous sentences, such as ‘fabricate’ for AM procedures, ‘heat treat’ for heat treatment, and ‘test’ for fatigue testing. Static mechanical properties such as Young’s modulus, yield strength (YS), ultimate tensile strength (UTS), and elongation are identified by REs in the paragraphs of the ‘Method’ and subsequent sections. The evaluation metrics of text classification and data extraction are summarized in Table 2. Both abstract and paragraph classification gain an F1 score higher than 80%. The F1 score of data extraction is 63%, which is not high since it is difficult to effectively introduce the context information in the rule-based RE approach. The processing of figures, tables, and text thus achieves good performance in the tasks of axis calibration, legend parsing, and text classification. The performance of data extraction can be improved by refining the parsing rules, employing dependency parsing, or using advanced NLP models such as the Generative Pre-trained Transformer (GPT). GPT-3 is a large pre-trained language model with 175 billion parameters with improved performance of few-shot learning^44^, which reduces the need for task-specific data and expertise in NLP. With fine-tuning, GPT-3 has the potential to extract structured data from complex scientific text with F1 score >80%^45^. The capability of GPT-4 is further elevated, especially in complex tasks^46^. Their applications to fatigue data remain to be explored.

### Database integration and data correction

To construct the database, fatigue data extracted from figures should be correlated with data entries of materials, AM, processing, testing, and static mechanical properties extracted from text and tables. Most of the data entries do not vary in specific research reported in an article. Single values extracted for a specific data entry are assigned to all datasets related to the article. For data entries with multiple values, the assignment is made according to the legend labels.

Unlike static mechanical properties, fatigue data are more sensitive to fabrication, processing, and testing conditions, resulting in data dispersion. Consequently, although the F1 scores of data extraction can be improved by using advanced techniques, the performance may still be insufficient to establish high-quality databases for fatigue analysis in engineering. In this work, we address this issue through manual examination and correction. For fatigue data, we firstly correct data using our IMEX interface, and then print out the data for comparison with those in the source figures. For entries related to materials, AM, processing, testing, and static mechanical properties, we export the data to an EXCEL file and compare them with the PDF files. Besides data examination and correction, the manual work also involves figure selection and segmentation, and legend region selection. We extract the size and shape of specimens during the manual examination since most of them are presented in figures instead of text. Examining the text is the dominant part of manual work, and a domain expert can process 4–8 articles per hour. An automated multimodal (figures, tables, and texts) data annotation and correction system could reduce the workload. Standardized data reporting coordinated by the authors, publishers, and data users can also facilitate the construction of databases.

### Fatigue data processing

In the experimental tests to measure the *S*-*N* and *ε*-*N* data, the amplitude (*σ*_a_ or *ε*_a_) and the maximum (*σ*_max_ or *ε*_max_) stress/strain are used, which can be related through

4 $${\sigma }_{{\rm{a}}}=\frac{{\sigma }_{{\rm{\max }}}-{\sigma }_{{\rm{\min }}}}{2}\quad {\rm{or}}\quad {\varepsilon }_{{\rm{a}}}=\frac{{\varepsilon }_{{\rm{\max }}}-{\varepsilon }_{{\rm{\min }}}}{2},$$

In the current study, the maxima (35% of the full database) are converted to amplitudes through the load ratio

5 $${R}_{\sigma }=\frac{{\sigma }_{{\rm{\min }}}}{{\sigma }_{{\rm{\max }}}}\quad {\rm{or}}\quad {R}_{\varepsilon }=\frac{{\varepsilon }_{{\rm{\min }}}}{{\varepsilon }_{{\rm{\max }}}},$$

6 $${\sigma }_{{\rm{a}}}=\frac{1-{R}_{\sigma }}{2}{\sigma }_{{\rm{\max }}}\quad {\rm{or}}\quad {\varepsilon }_{{\rm{a}}}=\frac{1-{R}_{\varepsilon }}{2}{\varepsilon }_{{\rm{\max }}}.$$

For the d*a*/d*N*-Δ*K* data, the SIF range is

7 $$\Delta K={K}_{{\rm{\max }}}-{K}_{{\rm{\min }}}.$$

For analysis, scattered fatigue data of the *S*-*N* and *ε*-*N* relations are fitted by assuming a log-normal distribution with a constant variance by following ASTM E739-10^47^, that is

8 $${\log }_{10}N=A+{B\log }_{10}{{\rm{\sigma }}}_{{\rm{a}}}\,{\rm{o}}{\rm{r}}\,{\log }_{10}N=A+{B\log }_{10}{{\rm{\varepsilon }}}_{{\rm{a}}},$$

where *A* and *B* are the fitting parameters. The *S*-*N* relation can be converted to the form of the Basquin’s equation

9 $${\sigma }_{{\rm{a}}}={A}_{1}{\left(N\right)}^{{B}_{1}},$$

where *A*_1_ and *B*_1_ are the fitting parameters.

The d*a*/d*N*-Δ*K* data are fitted by the Paris equation

10 $${\rm{d}}a/{\rm{d}}N=C{(\Delta K)}^{m},$$

where *C* and *m* are the fitting parameters.

## Data Records

**Fig. 4:**
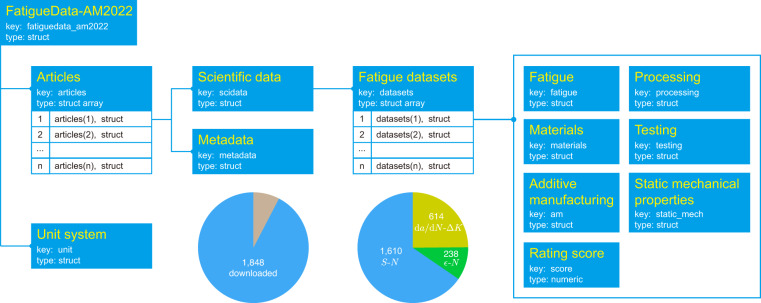
The structure of FatigueData-AM2022 database. The FatigueData-AM2022 database is formatted into a hierarchical tree structure. The name of each tree node is highlighted in yellow color. Keys are defined for easy access by scripts. Each node has its specific data type. Two pie charts show the statistics of downloaded articles and types of fatigue datasets.

The FatigueData-AM2022 database^48^ collects experimental *S*-*N*, *ε*-*N*, and d*a*/d*N*-Δ*K* data of AM alloys. The studies on structural components or architectured materials are not included^49,50^. Data are collected for fatigue tests under uniaxial or bending conditions. Fatigue performance under variable, torsional, and multiaxial loads are reported in only a few studies at this stage and are not incorporated to maintain data integrity. The FatigueData-AM2022 database^48^ is available as MAT (MATLAB), JSON, and EXCEL files at 10.6084/m9.figshare.22337629. The MAT and JSON files are formatted into a hierarchical tree structure. The tree nodes that directly store data values are called data entries. Data entries include string and numeric data types. Text data such as titles, types of AM, and fatigue tests are stored as strings. Data with multiple strings such as authors, countries, and institutions are stored as string arrays. The year of publication is defined as a numeric number, and other numeric data such as fatigue data, parameters of AM, and load ratios are stored in the form of numeric arrays. The tree nodes used to group data entries are called data structs. Multiple structs such as articles or fatigue datasets are arranged into struct arrays. To facilitate programming implementation and data acquisition, keys are defined for data entries, structs, and struct arrays (Fig. 4 and Tables 3–5).

**Table 3:** Contents of the struct of ‘metadata’ and children nodes of ‘fatigue datasets’.

Struct	Data Entry/Struct	Data Key	Data Type
**Metadata**			
	Title	title	string
	Authors	author	string array
	Source of the publication	source	string
	Year of publication	year	numeric
	Institution	institution	string array
	Country	country	string array
	Funding agency	fund	string array
	DOI	doi	string
**Fatigue**			
	Fatigue data	fat_data	numeric
	Types of fatigue data	fdata_type	string
	Method of extraction	extract_method	string
**Materials**			
	Name of the material	mat_name	string
**AM**			
	Types of AM	am_type	string
	AM parameters	am_para	struct
**Processing**			
	Processing parameters	proc_para	struct array
	Processing sequence	proc_seq	numeric
**Testing**			
	Types of fatigue tests	fat_type	string
	Fatigue temperature	fat_temp	numeric
	Fatigue environment	fat_env	string
	Load ratio	fat_r	numeric
	Frequency	frequency	numeric
	Fatigue machine	fat_machine	string
	Fatigue standard	fat_standard	string
	Specimens description	spec_desc	string
	Critical cross-section size of specimens	spec_size	numeric
	Stress concentration factor of specimens	spec_kt	numeric
	Load control	load_ctrl	string
**Static mechanical properties**			
	Young’s modulus	modulus	numeric
	Yield strength	yield_strength	numeric
	Ultimate tensile strength	tensile_strength	numeric
	Elongation	elongation	numeric

**Table 4:** Contents of the struct of ‘AM parameters’, dependent on the types of AM.

Types of AM	Data Entry	Data Key	Data Type
**For all**			
	AM machine	am_machine	string
	Direction of specimen	direction	numeric
	Scan speed	scan_speed	numeric
	Hatch space	hatch_space	numeric
	Layer thickness	layer_thickness	numeric
	Preheat temperature	preheat	numeric
	AM environment	am_env	string
	Layer scan rotation	layer_rot	numeric
	Scan pattern	scan_pattern	string
	Types of feedstock	fdstock_type	string
	Size of feedstock	fdstock_size	numeric
**Laser powder bed fusion (L-PBF)**			
	Power	power	numeric
**Electron beam powder bed fusion (E-PBF)**			
	Voltage	voltage	numeric
	Current	current	numeric
	Speed function	speed_func	numeric
**Powder-based directed energy deposition (P-DED)**			
	Power	power	numeric
	Voltage	voltage	numeric
	Current	current	numeric
	Powder feed rate	pfeed_rate	numeric
**Wire-based directed energy deposition (W-DED)**			
	Power	power	numeric
	Voltage	voltage	numeric
	Current	current	numeric
	Wire feed rate	wfeed_rate	numeric
**Others**			
	Power	power	numeric
	Voltage	voltage	numeric
	Current	current	numeric
	Wire feed rate	wfeed_rate	numeric
	Powder feed rate	pfeed_rate	numeric

**Table 5:** Contents of the struct in the ‘processing parameters’ struct array, dependent on the types of processing.

Types of processing	Data Entry	Data Key	Data Type
**For all**			
	Type	type	string
**Heat treatment (HT)**			
	Temperature	temperature	numeric
	Time	time	numeric
**Hot isostatic pressing (HIP)**			
	Temperature	temperature	numeric
	Time	time	numeric
	Pressure	pressure	numeric
**No heat treatment (NHT)**			
	–		
**Surface treatment (SURF)**			
	Method	method	string

The structure of the FatigueData-AM2022 database^48^ is summarized in Fig. 4. The root node is the database, containing children nodes of articles and a default unit system (e.g. MPa for stress, °C for temperature, μm for layer thickness, W for power). Raw numeric data are converted to the default units of data entries. Articles are stored as a struct array, and each article contains two structs of metadata and scientific data. Metadata contains data entries such as the titles and authors of articles. Scientific data store a struct array of fatigue datasets, each of which is obtained from experimental tests under different conditions. A fatigue dataset contains 6 structs (fatigue, materials, AM, processing, testing, and static mechanical properties), under which multiple data entries, structs, or struct arrays are defined (Table 3). A rating score is assigned to each fatigue dataset to measure the quality of data, which will be explained in the next section. The struct of AM parameters and processing parameters depends on their type, as shown in Tables 4, 5, respectively. The processing parameters are organized as a struct array, ‘proc_para’, for it may contain multiple steps. The processing sequence is recorded in the ‘proc_seq’ array. The processing parameters can be identified in the ‘proc_para’ array through the index entry in ‘proc_seq’.

The terminology of data types is largely inherited from MATLAB (the MAT file). Exceptions are string arrays and the struct array of processing parameters, which correspond to cell arrays in the MAT file. For the JSON file, the struct is defined as a dictionary, and all types of arrays are defined as lists. The FatigueData-AM2022 database^48^ is also flattened into an EXCEL file, including 4 worksheets. The worksheets of ‘S-N’, ‘e-N’, and ‘dadn’ store *S*-*N*, *ε*-*N*, and d*a*/d*N*-Δ*K* data, respectively. In these 3 worksheets, each row stores the index of a fatigue dataset and a data descriptor (*S*/*ε*, *N*, and the run-out flag for ‘S-N’/‘e-N’, d*a*/d*N* and Δ*K* for ‘dadn’). The d*a*/d*N*-Δ*K* data extracted by color stores all matched pixels. The number of data points exceeds the maximum number of rows allowed by EXCEL (1,048,576). As a result, 500 data points are sampled from each dataset and then recorded. In the 4th worksheet of ‘parameter’, each row stores the index of a fatigue dataset and its contents. Each column corresponds to a data entry. Data in the ‘parameter’ worksheet is linked to the other three through the index of fatigue datasets.

With the database structure outlined above, the data entries are explained here in detail. The ‘fatigue data’ array store *N* or Δ*K* in the first column, and the values of *σ*_a_, *ε*_a_ or d*a*/d*N* in the second column. *ε*_a_ stands for the amplitude of total strain including the elastic or plastic components. The third column stores the run-out flag for *S*-*N* and *ε*-*N* data, where ‘1’ denotes the test stops before failure (run-out) and ‘0’ denotes failure. The fatigue life and the FCG rate are sensitive to material anisotropy. In this work, the direction of specimens is measured by an angle between the building platform in AM and the loading direction^51^. The size effect of AM specimens could be significant due to the limited accuracy of printing, the presence of defects, and residual stress^52–54^. The size of the critical cross-section stores the diameter for specimens with circular cross-sections, the outer and inner diameters for those with annular cross-sections, and the width and thickness for those rectangular cross-sections, respectively. The shapes of the cross-sections are stored in the description of specimens (‘spec_desc’). In the numeric arrays of other data entries, a single value stands for a specific value or the mean, and two values stand for the lower and upper bound, respectively.

For the convenience of comparison between string data, unified nomenclature is used for data entries such as types of AM, materials, machines, affiliations, and funding agencies. 98% of the AM types can be classified into four categories of laser powder bed fusion (L-PBF), electron beam powder bed fusion (E-PBF), powder-based directed energy deposition (P-DED), and wire-based directed energy deposition (W-DED). Other AM types are recorded by their names such as binder jetting and metal extrusion. The default feedstock type is ‘powder’ for L-PBF, E-PBF, and P-DED, and ‘wire’ for W-DED.

In our database, data entries not reported explicitly are recorded as empty arrays (MAT), lists (JSON), strings (MAT and JSON), or cells (EXCEL). ‘As-built’ is assigned to surface treatment, ‘NHT’ is assigned to heat treatment, and ‘25 °C’ is assigned to preheat temperature if they are not applied (NA). We also assume that the testsing are uniaxial and conducted under an ambient environment (25 °C, air) with a stress concentration factor, *K*_t_ = 1 if not specified. The default load control is ‘force’ for *S*-*N*, ‘strain’ for *ε*-*N*, ‘load’ for d*a*/d*N*-Δ*K*, and ‘displacement’ for very high-cycle fatigue (VHCF) irrespectively of data types. It is suggested that optional procedures or settings should be stated as NA in reporting fatigue data if not specifically stated.

In summary, the FatigueData-AM2022 database^48^ covers 116 types of AM alloys in total. 459 articles report 1,610 *S*-*N* datasets with 15,146 data points, 79 articles report 236 *ε*-*N* datasets with 1,840 data points, and 135 articles report 614 d*a*/d*N*-Δ*K* datasets (Fig. 4). 65% of data are *S*-*N* data used to measure fatigue life in the HCF regime and for safe-life design^55–57^. Critical components in the aerospace and power industry under harsh conditions also require *ε*-*N* and d*a*/d*N*-Δ*K* data.

## Technical Validation

The performance metrics of figure, table, and text processing show that the F1 scores of automated extraction are ~60–90% (Table 2). All data records are manually examined and corrected to produce a high-quality database. Subsequent inspection of 50 randomly chosen articles shows that the precision is improved to be >98%.

One of the practical issues in extracting data from figures is the distortion of symbols and axis ticks after pixelation, which makes it difficult to determine the positions of centroids with high accuracy. Comparing *S*-*N* and *ε*-*N* data extracted from figures and those from the tables, if both of them were published, shows inconsistency in less than 5% of the 40 articles due to the uncertainties in locating the data points. The fitting parameters of data using Eq. 8 are compared with values reported in articles, also showing inconsistency <5%.

**Fig. 5:**
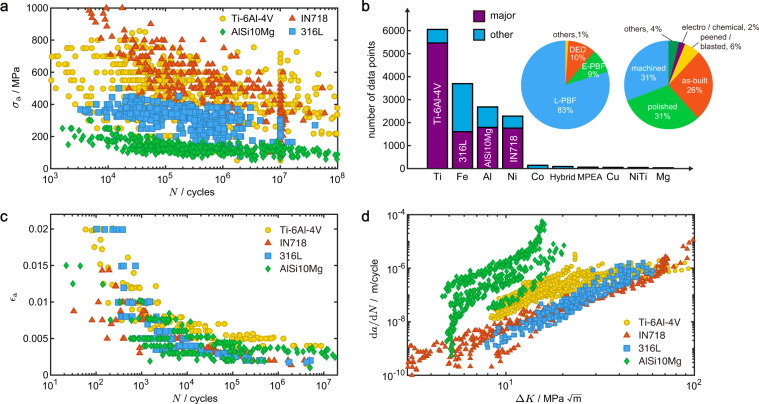
Representative data. (**a**) Representative *S*-*N* datasets of 4 major AM alloys, Ti-6Al-4V, IN718, 316 L and AlSi10Mg. (**b**) Statistics of AM alloys investigated for the *S*-*N* data. The *x*-axis is marked by the major element of alloys or their types. ‘MPEA’ denotes multi-principal element alloys. ‘Hybrid’ denotes hybrid or graded materials. The inset shows pie charts of types of AM and surface conditions, where ‘PBF’ denotes powder bed fusion, ‘L-PBF’ denotes laser PBF, ‘E-PBF’ denotes electron beam PBF, and ‘DED’ denotes directed energy deposition. Representative (**c**) *ε*-*N* and (**d**) d*a*/d*N*-Δ*K* data of major AM alloys.

Representative data and their statistics are plotted in Fig. 5 for illustration and the quality of data is assessed by the domain knowledge. *S*-*N* data for the 4 mostly reported AM alloys (Ti-6Al-4V, 316 L, AlSi10Mg, and IN718) are included in Fig. 5a and the fatigue life decreases as the stress amplitude increases. The fatigue strength of Ti-6Al-4V and IN718 alloys are superior, followed by 316 L and AlSi10Mg (Fig. 5a). The statistic of materials, types of AM, and surface treatment of *S*-*N* datasets are summarized in Fig. 5b. Ti-6Al-4V occupies 90% of the data for AM titanium alloys, and IN718 occupies 77% for AM nickel alloys. The high percentage of occupations stems from their dominance in conventional titanium and nickel alloys for the high strength and mature manufacturing procedures^58,59^. Though AlSi10Mg is not very popular among conventional aluminum alloys, it accounts for 66% of AM aluminum alloys due to its good printability^60^. 316 L accounts for only 43% of AM steels and other types also take a share, signaling the diversity in the applications of steels^61^. It is noted that most of the fatigue specimens are prepared by PBF, especially L-PBF (83%), which is the most mature and commercialized AM technique (Fig. 5b)^61^. The layer-by-layer printing process and non-equilibrium nature of AM may result in poor surface quality, to which the *S*-*N* data are susceptible. Different types of surface treatment are investigated (Fig. 5b).

**Fig. 6:**
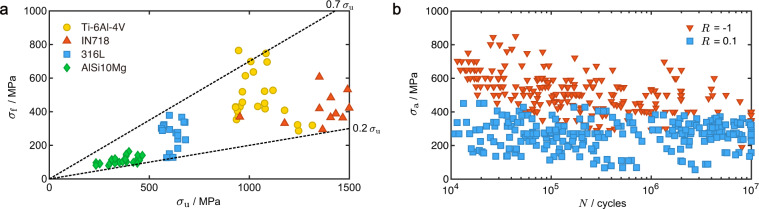
Data validation. (**a**) Relation between fatigue strength measured after 10^6^ cycles, *σ*_f_, and ultimate tensile strength (UTS), *σ*_u_. References *σ*_f_ = 0.2*σ*_u_ and *σ*_f_ = 0.7*σ*_u_ are added as the dashed lines. (**b**) The effect of the stress ratio, *R*, on the *S*-*N* relations of AM Ti-6Al-4V.

Representative *ε*-*N* and d*a*/d*N*-Δ*K* data are shown in Fig. 5c, d. The fatigue life decreases as strain amplitude increases (Fig. 5c), and the FCG rate increases with the SIF range (Fig. 5d). The quality of data is further assessed by the relationship between fatigue data and other properties of the alloys, which is demonstrated here using the *S*-*N* data as an example. The relation between fatigue strength (*σ*_f_) and UTS (*σ*_u_), and the effects of loading and processing conditions are well-known for conventional alloys^2,62,63^. Fig. 6a confirms the positive correlation between *σ*_f_ and *σ*_u_, that is, high *σ*_u_ indicates high resistance to fatigue by suppressing damage accumulation. The ratio between *σ*_f_ and *σ*_u_ (0.2–0.7) for AM alloys is close to that of conventional alloys (0.25–0.65)^2^.

*S*-*N* tests are commonly conducted at specific stress ratios, *R*_*σ*_, which could introduce the effect of mean stress, *σ*_m_ = (*σ*_max_ + *σ*_min_)/2. The relation between *R*_*σ*_ and *σ*_m_ can be derived from Eq. 5, which is ${R}_{\sigma }=1-\frac{2{\sigma }_{{\rm{a}}}}{{\sigma }_{{\rm{m}}}+{\sigma }_{{\rm{a}}}}$. Figure 6b shows the performance of AM Ti-6Al-4V tested under *R*_*σ*_ = −1 (*σ*_m_ = 0) and 0.1 (*σ*_m_ = 0.55σ_max_). The mean tensile stress downgrades the fatigue strength even under strong data dispersion, which agrees with the domain knowledge of conventional alloys as well.

**Fig. 7:**
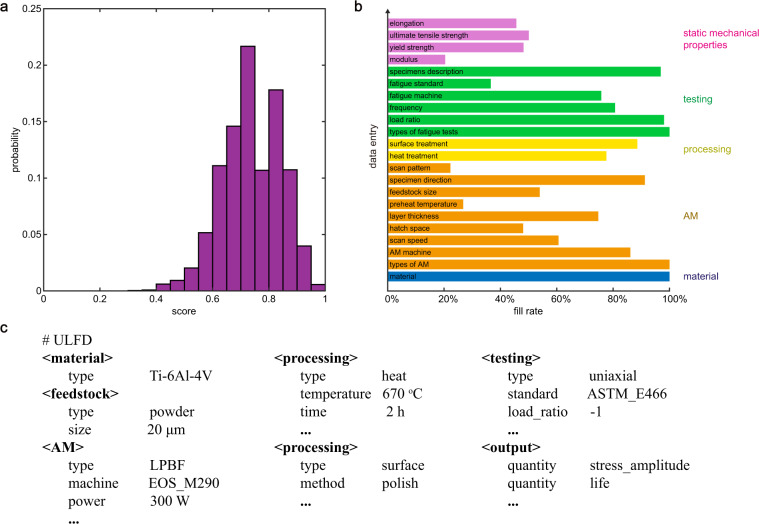
Data quality measured by rating scores and fill rates. (**a**) The histogram of the rating scores for the fatigue datasets, where all of the data entries are equally weighted. (**b**) The fill rates (FRs) of the types of materials, parameters of AM, processing, testing, and static mechanical properties. (**c**) The unified language of fatigue data (ULFD).

There are limitations in the applications of fatigue databases constructed from open sources in comparison with the datasets released from authoritative institutions. In addition to the diversity in material fabrication, sample preparation, and surface finishing of the specimens, the incompatibility in testing standards and incompleteness of records also lead to difficulties in improving the quality of data, as well as the integration with authoritative databases or new data reported in the literature. A rating system is introduced for the data to be used in the design of structural integrity. Data entries can be assigned with weights according to the domain knowledge or their covariance with fatigue data. Additional measures such as the number of fatigue data^47^, the number of citations of the publication, and the accuracy of data extraction could also be introduced. For each fatigue dataset, a rating score between 0 and 1 is computed as the weighted summation of non-empty entries. The scoring algorithm is subjective, and we leave this work to data users. Here, for the sake of simplicity, we assume equal weights for all the entries (Fig. 7a). Surface and heat treatment (including HIP and NHT) are regarded as two separate entries of processing parameters. We find that most datasets are rated with scores ranging from 0.5 to 0.9 since not all of the data entries are documented. 87% of the datasets have scores higher than 0.6, which contain essential information such as types of materials, types of AM, and fatigue testing. Fill rates (FRs) of data entries counted over all the datasets measures the quality of the database (Fig. 7b), which is expected to be not high for the diversity of data sources. The types of materials (e.g. Ti-6Al-4V, IN718), AM (e.g. PBF, DED), fatigue testing (e.g. uniaxial, bending), and load ratios are essential information and are provided in most AM fatigue articles. For the data entries related to AM and processing, the FRs of AM machine, layer thickness, the direction of specimens, heat treatment, and surface treatment are higher than 70% whereas other entries are less filled. For fatigue testing, 80% articles reported the loading frequency since it could vary by 4 orders of magnitudes in practice. The effects of frequency could be significant as the heating effect is introduced, for example, by plastic dissipation in LCF or vibration in VHCF. In addition, the strain rate is proportional to the frequency, to which the damage processes could be susceptible, and in a corrosive environment, material degradation is rate-dependent as well^64,65^. Surprisingly, only 40% articles reported the standard of fatigue testing they followed. Considering the variation in microstructures and (as-built) surface conditions, the implementation of traditional fatigue testing standards for AM fatigue research should be assessed^66^. New designs of specimens, e.g. in miniature types^67^, and testing techniques such as VHCF are also worth further discussion. FRs of static mechanical properties are no more than 50% since the data dispersion is not high.

Our results highlight the need for standards of AM fatigue testing as well as norms of reporting data in journals, conference proceedings, and technical reports, which are crucial for the development of high-quality databases and data-centric research. A unified language of fatigue data (ULFD) is suggested here according to related standards for AM, processing, and testing^68^. The current database can be exported using the ULFD (Fig. 7c), which not only outlines the workflow of database construction but also guides data analysis and experimental planning.

## Usage Notes

**Fig. 8:**
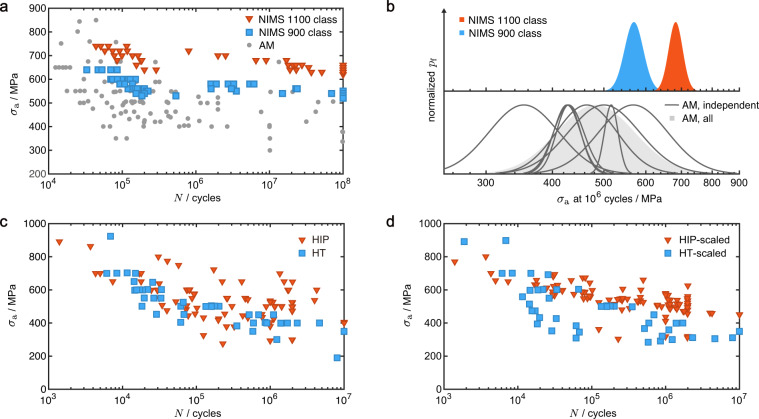
Data dispersion and system deviation. (**a**) The comparison between the *S*-*N* data of AM alloys extracted from the literature and the NIMS data released for Ti-6Al-4V under the stress ratio *R* = −1. ‘1100 class’ indicates that the UTS is on the level of 1100 MPa and ‘900 class’ indicates 900 MPa. (**b**) The probability density function, *p*_f_, of fatigue strength, *σ*_f_, after 10^6^ cycles for datasets in (**a**), normalized by the maxima. The NIMS data are shown in the upper panel and AM data in the lower panel. For the AM data, lines indicate the data fitted from independent datasets, and the shaded area collects all the data. (**c**) The comparison between the *S*-*N* data for heat-treated (HT) and hot isostatically pressed (HIP) Ti-6Al-4V alloys. The datasets are scaled in (**d**) according to the reference data (Eq. 11).

Data dispersion and system deviation should be noted while analyzing fatigue data reported in the literature. For example, the fatigue strength of AM Ti-6Al-4V is not only inferior to its conventional counterpart as reported in the NIMS database but also shows a larger scatter (Fig. 8a). Comparison to the MMPDS data leads to the same conclusion. To quantify the degree of dispersion, the log-normal probability density function ${p}_{{\rm{f}}}\left(x\right)=\frac{1}{s\sqrt{2\pi }}\exp \left[-\frac{1}{2}{\left(\frac{ln(x)-\mu }{s}\right)}^{2}\right]$ is assumed and fitted using Eq. 9 to compute the mean, *μ*, and variance, *s* of the fatigue strength after 10^6^ cycles (Fig. 8b). The values of *s* for the datasets range from 1.6 × 10^−4^ to 45.1 × 10^−4^, most of which are higher than the values in NIMS 1100 class (3.1 × 10^−4^) and 900 class for Ti-6Al-4V (1.7 × 10^−4^). AM data are more scattered than the NIMS data regardless of the types of materials, which can be attributed to the diversity in material microstructures including the defects. Optimizing AM parameters or post-processing procedures could reduce the dispersion of fatigue performance and better serve critical applications. Although displaying a more scattered nature compared to authoritative databases, AM data collected from the literature still provide key insights into the material properties and guidelines for fatigue design (Fig. 5).

In addition to data dispersion, Fig. 8b shows that system deviation exists among fatigue data from different studies. For example, hot isostatic pressing (HIP) is an effective high-pressure, high-temperature procedure to reduce internal (porous) defects in alloys, which improves their HCF performance by suppressing crack initiation. The effect of HIP on fatigue performance is compared to that of ordinary heat treatment that operates at lower temperatures without pressurization (Fig. 8c). The two sets of data can hardly be distinguished due to not only data dispersion, but also system deviation resulting from differences in the specimen preparation and testing procedures. To resolve this issue, one of the published HIP fatigue data is selected as a reference. All HIP fatigue data are then fitted by Basquin’s equation (Eq. 9) and scaled to the reference. The scaling factor for *σ*_a_ at specific cycles *N* is calculated as

11 $$\alpha =\frac{{A}_{1}^{{\rm{ref}}}{\left(N\right)}^{{B}_{1}^{{\rm{ref}}}}}{{A}_{1}{\left(N\right)}^{{B}_{1}}},$$

where the superscript ‘ref’ denotes the reference data. The heat treatment (HT) data are then scaled using the value of *α* for the HIP data reported in the same articles, that is, ${\sigma }_{{\rm{a}}}^{{\rm{HT}},{\rm{scaled}}}=\alpha {\sigma }_{{\rm{a}}}^{{\rm{HT}}}$. The results clearly show that HIP outperforms HT in improving the HCF performance, where fatigue life is controlled by crack initiation (Fig. 8d). However, HT seems to be superior for LCF (*N* < 10^4^), where plastic deformation is crucial. This can be explained by the process of grain coarsening in HIP, which weakens the resistance of alloys to plastic deformation^69^.

Our database lays the ground for data-driven material screening and life estimation of AM components, offering cost-effective solutions for engineering design. Critical analysis of the entries in the database offers key insights into technical roadmapping^70^, which could optimize the investment strategy in research and development. Our database can also serve as a training dataset for NLP, ML, and CV models to improve the performance of model predictions. In addition, the current approach can be extended to other information on AM alloys and fatigue data of other alloys. However, extracting data from earlier literature for conventional alloys could suffer from challenges in processing image-based PDFs, where both text and figures/tables are of low quality and difficult to extract. Future work will focus on improving the level of automation of the current workflow and addressing the problems of parsing early documents.

## Data Availability

The scripts utilized to extract information from figures, tables, and text are mainly based on open-source codes such as ChemDataExtractor 2.0^43^, table extractor^39^, and Simple Transformer (https://simpletransformers.ai/), respectively. The in-house scripts for data extraction and analysis are publicly released at the GitHub repository (https://github.com/xuzpgroup/ZianZhang/tree/main/FatigueData-AM2022), which can be used by acknowledging the current article and under the MIT license^71^. These scripts include a detailed, step-by-step tutorial for loading and analyzing the dataset in the repository.
